# Plyometric Training Favors Optimizing Muscle–Tendon Behavior during Depth Jumping

**DOI:** 10.3389/fphys.2017.00016

**Published:** 2017-01-25

**Authors:** Kuniaki Hirayama, Soichiro Iwanuma, Naoki Ikeda, Ayumi Yoshikawa, Ryoichi Ema, Yasuo Kawakami

**Affiliations:** ^1^Faculty of Sport Sciences, Waseda UniversityTokorozawa, Japan; ^2^Department of School Education, Teikyo University of ScienceTokyo, Japan; ^3^Graduate School of Sport Sciences, Waseda UniversityTokorozawa, Japan; ^4^Research Fellow of Japan Society for the Promotion of ScienceTokyo, Japan; ^5^Graduate School of Engineering and Science, Shibaura Institute of TechnologySaitama, Japan

**Keywords:** muscle–tendon interaction, fascicle, ultrasonography, electromyography, magnetic resonance imaging

## Abstract

The purpose of the present study was to elucidate how plyometric training improves stretch–shortening cycle (SSC) exercise performance in terms of muscle strength, tendon stiffness, and muscle–tendon behavior during SSC exercise. Eleven men were assigned to a training group and ten to a control group. Subjects in the training group performed depth jumps (DJ) using only the ankle joint for 12 weeks. Before and after the period, we observed reaction forces at foot, muscle–tendon behavior of the gastrocnemius, and electromyographic activities of the triceps surae and tibialis anterior during DJ. Maximal static plantar flexion strength and Achilles tendon stiffness were also determined. In the training group, maximal strength remained unchanged while tendon stiffness increased. The force impulse of DJ increased, with a shorter contact time and larger reaction force over the latter half of braking and initial half of propulsion phases. In the latter half of braking phase, the average electromyographic activity (mEMG) increased in the triceps surae and decreased in tibialis anterior, while fascicle behavior of the gastrocnemius remained unchanged. In the initial half of propulsion, mEMG of triceps surae and shortening velocity of gastrocnemius fascicle decreased, while shortening velocity of the tendon increased. These results suggest that the following mechanisms play an important role in improving SSC exercise performance through plyometric training: (1) optimization of muscle–tendon behavior of the agonists, associated with alteration in the neuromuscular activity during SSC exercise and increase in tendon stiffness and (2) decrease in the neuromuscular activity of antagonists during a counter movement.

## Introduction

It is known that plyometric training improves exercise performance that involves stretch–shortening cycle (SSC) of muscle–tendon units (MTU) (Markovic and Mikulic, 2010), but the underlying mechanisms are not adequately elucidated. An MTU with a long tendon (e.g., the triceps surae) displays unique behavior during SSC; muscle fascicles experience relatively small length changes, while the tendon covers the majority of length changes (stretch–shortening) of the MTU (Fukunaga et al., 2001; Hirayama et al., 2012). The magnitude of power or work generated by the MTU during the SSC as such, would depend on the muscular strength and tendon stiffness, as well as on the neuromuscular activity throughout the movement. Most previous studies delineating the mechanism by which plyometric training improves SSC exercise performance have focused on the changes in muscle strength and tendon stiffness (Burgess et al., 2007; Kubo et al., 2007b; Fouré et al., 2010; Wu et al., 2010) and/or neuromuscular activity (Toumi et al., 2004; Kubo et al., 2007b). To the best of our knowledge, no study has ever elucidated how muscle–tendon behavior during the SSC changes in response to long-term plyometric training.

Hirayama et al. (2012) showed that a single-session, plyometric exercise alters the muscle–tendon behavior through modulation of neuromuscular activity with the tendon undergoing more lengthening and shortening compared with the fascicles. Unlike such an acute effect of a particular exercise, long-term plyometric training would improve SSC exercise performance differently, with the MTU behavior changing into a favorable way, matching altered muscular strength and tendon stiffness (Burgess et al., 2007; Fouré et al., 2010; Wu et al., 2010).

In the present study, we aimed to elucidate the mechanisms behind how plyometric training improves SSC exercise performance with changes in muscle strength, tendon stiffness, and muscle–tendon behavior. Considering that plyometric training enables one to jump higher with shorter ground contact time (Markovic and Mikulic, 2010), it was hypothesized that the changes in neuromuscular factors are associated with muscle force generated by the agonists during SSC exercise. In addition, we hypothesized that tendon stiffness would increase as a result of plyometric training, thereby leading to faster tendon recoil after being elongated and to improvement of SSC exercise performance. We also hypothesized that such a change in tendon is accompanied by a more quasi-isometric contraction of the muscle fascicles so that they can exert greater force compared to concentric contraction.

## Materials and methods

### Subjects

Twenty-one recreationally active males participated in this study. They did not have any habit that includes plyometric exercise. The participants were randomly assigned to two groups: the training group (*n* = 11; age: 22 ± 3 years, height: 172.0 ± 5.8 cm, weight: 66.9 ± 10.5 kg, mean ± SD) and the control group (*n* = 10; age: 22 ± 4 years, height: 174.5 ± 5.4 cm, weight: 66.7 ± 8.0 kg). There were no significant differences between the training and control groups with respect to age, height, or weight. All subjects were informed of the purpose, experimental procedure, potential risk of this study and their right, and gave their written consent to participate. The study was approved by the Ethics Review Committee on Human Research of Waseda University.

### Plyometric training program

**Figure 1:**
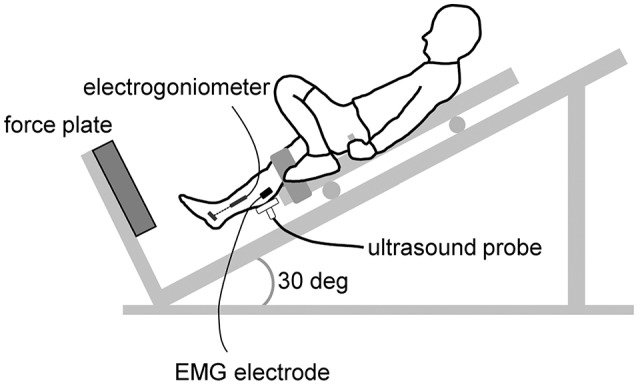
**Schematic presentation of the depth jumping during the test measuremants**. During the training session, the ultrasound probe, EMG electrode, and electrical goniometer were removed.

The training group completed plyometric training for 12 weeks (3 days/week), with a rest period of 1 week halfway through the training period. For one session of plyometric training, the subjects repeated depth jumps (DJ) for 10 sets of 10 repetitions with a rest interval of 30 s. We defined a DJ as a unilateral single-joint plantar flexion from a certain height on a sledge apparatus (Inclined Squat, Vine, Tokyo, Japan) (Figure 1). The subjects lay in the supine position on the sliding bed of the sledge apparatus, which was inclined 30° from the floor. The knee of the right leg was secured to the sliding bed with non-elastic straps and a solid pad set behind the knee, in a slightly flexed position to minimize excessive strain on the knee joint. The left leg was rested on the bed with the knee in a flexed position. The sledge apparatus was set up to start falling from a preset position when the subject let go of the harness handle that held the bed in position. The drop height (14.3 ± 1.8 cm) was determined according to the lower leg length of each subject, and the height was kept constant over the experimental period for each subject. In the DJ, the subjects landed on a force plate (9281B, Kistler, Winterthur, Switzerland) attached to the sledge apparatus with the ball of foot. Thereafter, the subject voluntarily arrested the falling motion by eccentrically plantar flexing the ankle (braking phase). The ankle was then plantar flexed to perform propulsive movement (propulsive phase). The subjects were requested to jump as high as possible. The control group was requested to maintain their daily activities during the period; they were allowed to carry on with their own exercise habits, but none of them had experience in plyometric training before and during the present study.

### Measurements

Before (Pre) and after (Post) the training or control period, muscle strength, tendon stiffness, and DJ performance were quantified. Before the measurements, a standardized warm-up (i.e., slow and fast unilateral calf raise and static plantar flexion) was performed.

### Muscle strength and tendon stiffness

The subjects lay on the myometer (VTF-002, Vine, Tokyo, Japan) in the prone position with the knee fully extended. Then, they performed static plantar flexion with maximal effort at an ankle angle of 0° (anatomical position). This measurement was repeated twice, and the highest value of torque was adopted.

The Achilles tendon stiffness was quantified using ultrasonography (ProSound α7, Hitachi Aloka Medical, Ltd., Tokyo, Japan). The ultrasound probe (scan width: 60 mm; UST-5713T, Hitachi Aloka Medical, Ltd., Tokyo, Japan) was attached to the skin with a foam pad and adhesive tapes over the mid-belly of the medial gastrocnemius, to capture ultrasonic images of the medial gastrocnemius during torque development at 48 Hz. The images were electrically synchronized with other data. The subject developed static torque by gradually increasing torque from zero (relaxed state) to the maximum over approximately 5 s. The measurement was repeated at least twice per subject, and the trial with a smooth torque trace up to the highest value was selected for analysis. The displacement of the intersection of a clearly visible fascicle and deep aponeurosis was measured using a software (Image J, National Institutes of Health, Bethesda, MD, USA) and was defined as the elongation of the Achilles tendon (Hansen et al., 2003; Kubo et al., 2015). To correct for slight ankle displacement during plantar flexion that inevitably occurred during data collection, an additional measurement was made in which the ankle was passively plantar flexed. And, the relationship between the ankle angles and the movement of the fascicle–aponeurosis intersection was determined. The amount of intersection movement corresponding to the ankle displacement observed in the tendon stiffness test was later deducted. The elongation of the tendon was synchronized with the force every 10% of peak torque from rest to peak torque. The force was calculated by dividing torque by the moment arm of the Achilles tendon (section “The Achilles tendon moment arm” later). The slope of this relation from 50 to 100% peak torque was defined as the tendon stiffness (Kubo et al., 1999).

### Kinetics and kinematics of DJ

**Figure 2:**
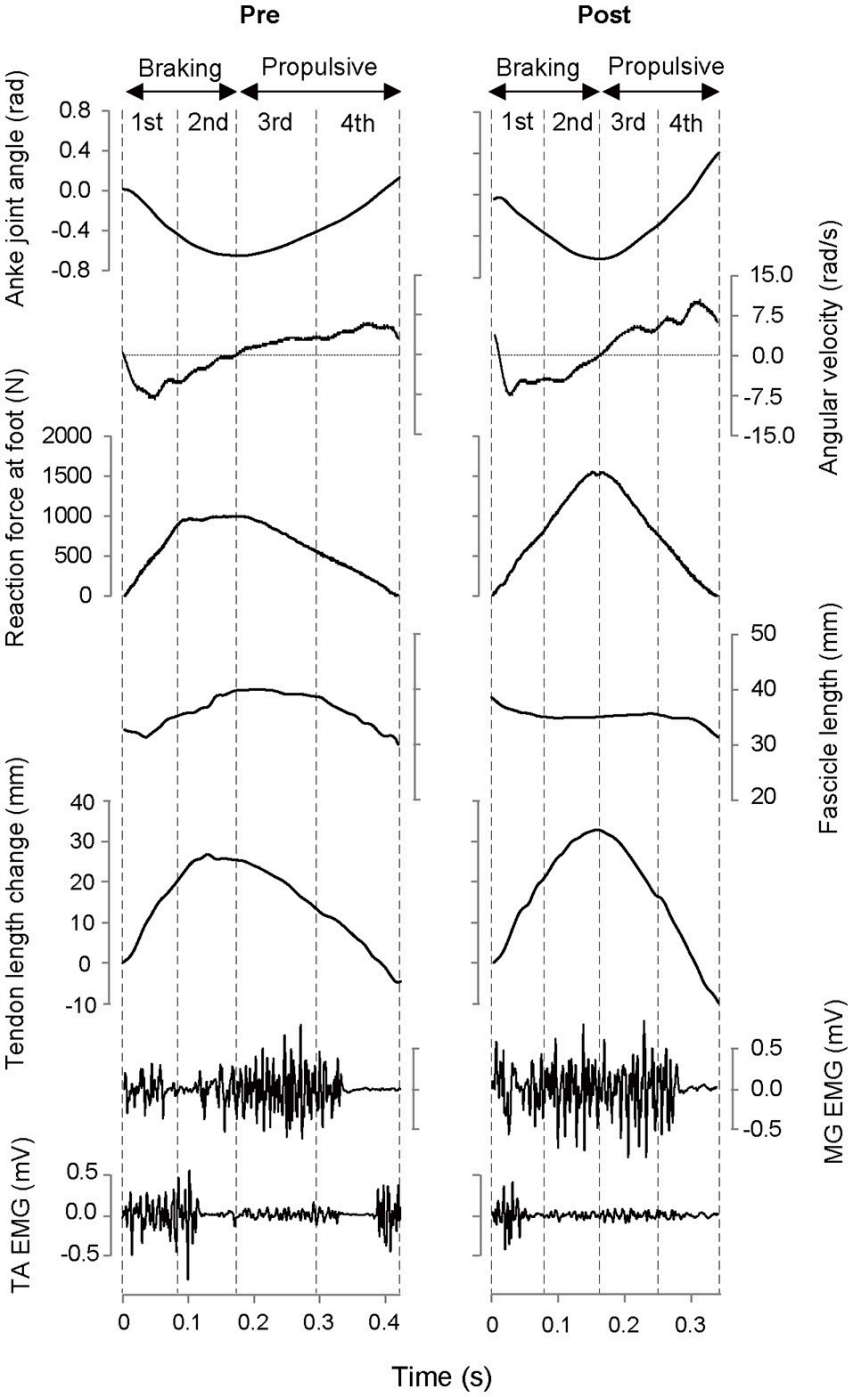
**Typical example of time course of measured variables during depth jump in the Pre- and Post-test for the training group**. MG, medial gastrocnemius muscle; TA, tibialis anterior muscle.

The subjects performed DJ for 2 or more repetitions. During the DJ, the kinetic and kinematic parameters were recorded (Figures 1, 2).

The signals of the component of the reaction force at the foot perpendicular to the surface of the force plate were amplified and A/D converted at a sampling rate of 2 kHz (PowerLab/16 sp, ADInstruments, Bella Vista, Australia). This was then transmitted to a computer. The force measured at rest was set as the baseline (i.e., offset to zero) and integrated until the force dropped back to the baseline to determine the impulse. A trial with the largest impulse was used for later analyses.

The ankle joint angle (dorsi- and plantar flexion) during DJ was recorded with an electrogoniometer (Twin Axis Goniometers SG110/A, Biometrics Ltd, Newport, UK) attached to the skin over the medial side of the tibia and rear foot. The ankle joint angle was defined as the angle between the tibia and plantar surface. The electrogoniometer was calibrated using a myometer for each subject. The output signals were low-pass filtered at 30 Hz to reject slight noise made by the AC source and A/D converted at a sampling rate of 2 kHz.

Neuromuscular activities during DJ were measured by surface electromyography (EMG) with Delsys EMG data acquisition system (Bagnoli-8, Delsys Inc., Boston, MA, USA). Before attaching electrodes to the skin, skin preparation was performed by shaving, abrading, and cleaning with alcohol. Active surface electrodes (interelectrode distance of 10 mm, DE-2.1, Delsys Inc., Boston, MA, USA) were placed on the skin over the mid-belly of the medial gastrocnemius, lateral gastrocnemius and the tibialis anterior. Electrodes were also placed over the lateral aspect of the soleus along the longitudinal axis of each muscle. The EMG signals were amplified and A/D converted at a sampling rate of 2 kHz. The EMG amplitudes were rectified and averaged for the following movement phases (mEMG): 1st phase (initial half of the braking phase), 2nd phase (second half of the braking phase), 3rd phase (initial half of the propulsive phase), and 4th phase (second half of the propulsive phase). The mEMG value in each phase was normalized to those during the maximal isometric contraction. The mEMG value of the medial and lateral gastrocnemii and soleus were averaged to determine mEMG of the triceps surae. For the training group, EMG electrode attachment sites were marked on the skin with an indelible pen, which were over-written on every occasion of the training session.

Longitudinal sectional images of the medial gastrocnemius during DJ were captured at 99 Hz with the ultrasound apparatus. The fascicle length (length of the fascicular path between the insertions of the fascicle into the superficial and deep aponeuroses) and pennation angle (angle formed by a fascicle and deep aponeurosis) of the medial gastrocnemius were measured using a digitizing software (Hirayama et al., 2012). One pixel of the digitized ultrasound image represented 0.15 × 0.15 mm. The coefficients of variation were 1.7% for fascicle length and 2.1% for pennation angle when the analyses were repeated twice on the same images. Averaged fascicle length and contraction velocity were calculated for each DJ phase.

The tendon length change in the medial gastrocnemius was determined by subtracting the change in MTU length from the “effective fascicle length change” of the muscle (Fukunaga et al., 2001; Spanjaard et al., 2007; Hirayama et al., 2012). The length change of the gastrocnemius MTU was calculated as the product of the Achilles tendon moment arm and ankle angle change in radians (Chino et al., 2008). The “effective fascicle length change,” which represents the length change in the longitudinal direction of the muscle belly, was obtained by multiplying fascicle length by the cosine of the pennation angle (Chino et al., 2008). Velocity of the tendon length change was calculated by differentiating the tendon length change, and this was averaged over each DJ phase.

### The achilles tendon moment arm

The Achilles tendon moment arm was determined from the magnetic resonance (MR) images with the center of rotation method described by Maganaris et al. (1998). A series of sagittal MR images of the ankle joint were obtained at a 10° dorsi-flexed position, neutral position, and 10° plantar flexed position, with the lower leg muscles relaxed. The MR imaging was performed using a 1.5-T MR imaging scanner (Signa HDxt, GE Healthcare, Milwaukee, WI, USA) with a proton density weighted fast recovery fast spin echo sequence (TR: 1300 ms, TE: 20 ms, slice thickness: 5 mm, interspaced distance: 0 mm, FOV: 260 × 260 mm, matrix: 256 × 160 pixels). The number of slices was set to cover the width of the calcaneus, talus, and tibia (i.e., 14–15 slices). The center of rotation in the talocrural joint at the neutral foot position was calculated from the MR images at a 10° dorsi-flexed position and 10° plantar flexed position using the Reuleaux method (Reuleaux, 1876). The moment arm was determined as the perpendicular distance from the center of rotation of the talocrural joint to the line of action of the Achilles tendon force at a neutral foot position.

### Statistical analysis

Descriptive data are expressed as mean ± SD. A two-way analysis of variance (ANOVA) with repeated measures (group [training and control] × time [Pre and Post]) was used to determine the effects of training on the strength, tendon stiffness and impulse and contact time of DJ. When appropriate, the Bonferroni *post-hoc* test was performed to examine the difference between training group and control group or Pre-test and Post-test. A two-way ANOVA with repeated measures (time [Pre and Post] × phase [1st, 2nd, 3rd, and 4th or braking and propulsive]) was used to test the kinetic and kinematic parameters, which were determined for each DJ phases. When appropriate, we performed the Bonferroni *post-hoc* test to examine the difference between Pre- and Post-test. To define the contraction mode of the fascicle during each phase of DJ, one sample *t*-test was performed for fascicle contraction velocity: when a significant difference from zero was observed, the contraction mode was defined as eccentric or concentric; otherwise the manner was defined as isometric. The level of significance was set at *p* < 0.05.

## Results

We failed to obtain the ankle joint angle during DJ from three subjects, and the reaction force, muscle–tendon behavior and EMG data for training group are presented as averages for eight subjects. Similarly, failure to obtain the ankle joint angle for one subject resulted in nine subjects being included in the control group.

### Strength and tendon stiffness

Table 1 shows measurement variables of the strength and tendon stiffness. At the Pre-test (baseline), there were no significant differences between the training and control groups in static plantar flexion torque or tendon stiffness. For the training group, the tendon stiffness increased significantly (*p* = 0.017) after the training period, while isometric plantar flexion torque did not change. For the control group, there were no significant differences between Pre- and Post-test sessions for these variables.

**Table 1:** **Muscle strength and tendon stiffness measured under static conditions**.

	**Training group**		**Control group**	
	**Pre**	**Post**	**Pre**	**Post**
Static plantar flexion torque (Nm)	149 ± 16	153 ± 19	146 ± 19	146 ± 13
Achilles' tendon stiffness (N/mm)	193 ± 52	260 ± 67^*^	203 ± 59	185 ± 79

* *p < 0.05*.

### DJ performance and kinetics

**Figure 3:**
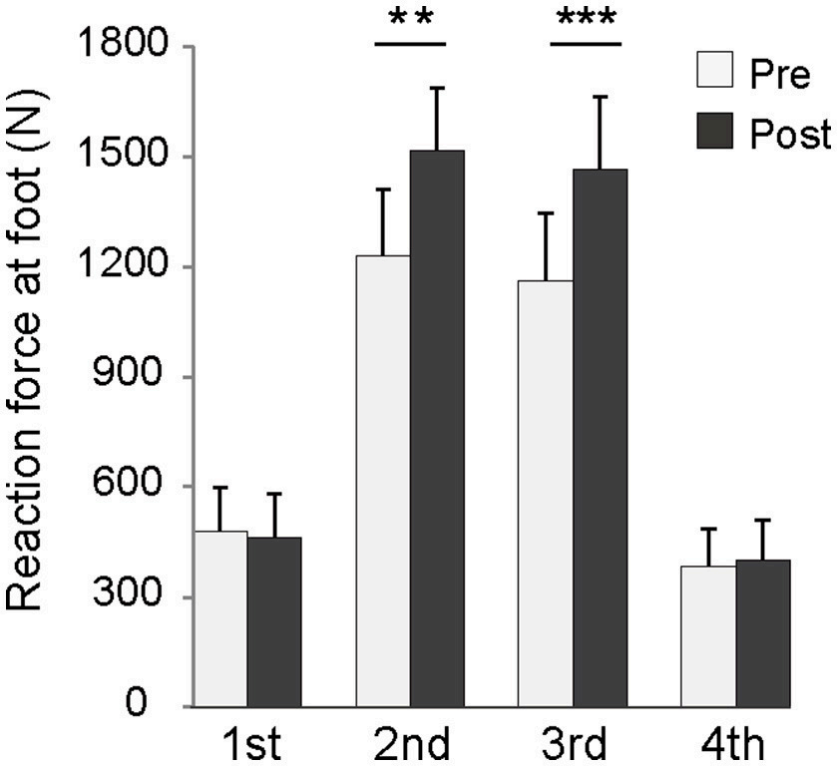
**Averaged reaction force in each phase of depth jump for the training group**. ^**^p < 0.01, ^***^p < 0.001.

No significant difference between the groups in impulse during DJ was found at Pre-test (baseline). The impulse during DJ increased significantly for the training group (Pre: 168 ± 21 N·s, Post: 192 ± 20 N·s) (*p* < 0.001) but not for the control group (Pre: 160 ± 13 N·s, Post: 155 ± 20 N·s). For the training group, the contact time of DJ decreased significantly (Pre: 0.365 ± 0.068 s, Post: 0.310 ± 0.043 s) (*p* = 0.010), and the averaged reaction force during the 2nd and 3rd phases increased significantly (Figure 3). For the control group, there were no significant changes in the contact time (Pre: 0.388 ± 0.061 s, Post: 0.402 ± 0.093 s) and averaged reaction force (1st phase: 451 ± 79 N [Pre], 426 ± 98 N [Post], 2nd phase: 1111 ± 205 N [Pre], 1141 ± 253 N [Post], 3rd phase: 1054 ± 152 N [Pre], 1117 ± 201 N [Post], and 4th phase: 391 ± 52 N [Pre], 346 ± 67 N [Post]).

### Muscle–tendon behavior during DJ

**Figure 4:**
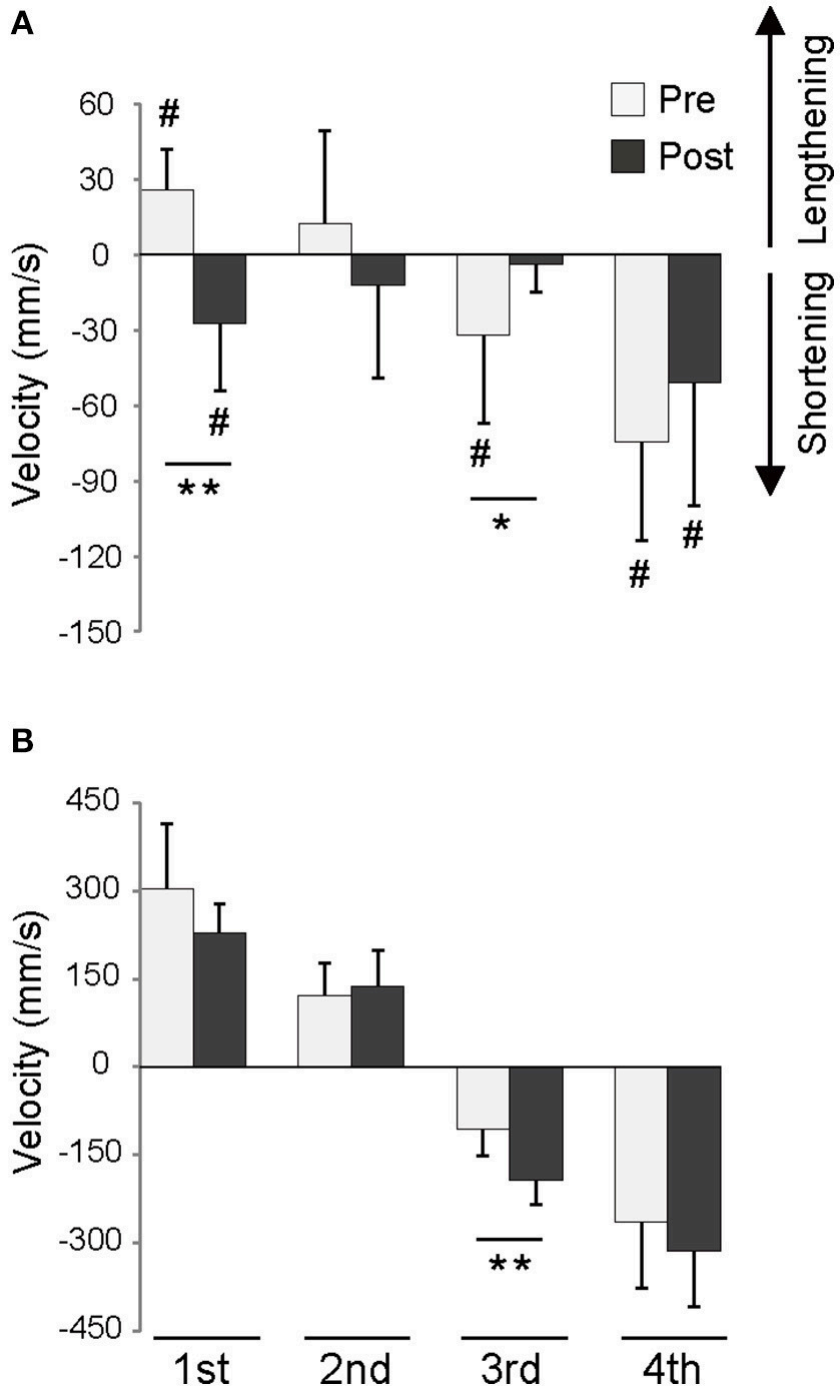
**Velocity of fascicle (A)** and tendon **(B)** during each phase of depth jump for the training group. ^*^p < 0.05, ^**^p < 0.01, ^#^significant difference from zero (*p* < 0.05).

Figure 4A shows the velocity of the fascicle at each phase of DJ for the training group. In the 1st phase, contraction mode was eccentric in the Pre-test but became concentric in the Post-test. The fascicle contracted isometrically during the 2nd phase both in Pre-test and Post-test, and contraction velocity exhibited no significant difference between Pre-test and Post-test. In the 3rd phase, shortening velocity of the fascicle decreased significantly, while contraction mode was concentric in the Pre-test and slowed to isometric in the Post-test. The fascicle contracted concentrically during both Pre-test and Post-test in the 4th phase, with no significant difference in contraction velocity. In the control group, no differences were observed in fascicle contraction velocity between the Pre-test and Post-test (1st phase: 4 ± 55 mm/s [Pre], 15 ± 55 mm/s [Post], 2nd phase: 29 ± 38 mm/s [Pre], 22 ± 28 mm/s [Post], 3rd phase: −29 ± 17 mm/s [Pre], −33 ± 23 mm/s [Post], and 4th phase: −107 ± 42 mm/s [Pre], −109 ± 54 mm/s [Post]). Averaged fascicle length before and after training did not differ in any of the phases for either the training or the control group (Table 2).

**Table 2:** **Averaged fascicle length during depth jump**.

		**Training group**		**Control group**	
		**Pre**	**Post**	**Pre**	**Post**
Fascicle length (mm)	1st phase	49 ± 11	52 ± 9	51 ± 11	52 ± 14
	2nd phase	51 ± 10	51 ± 8	54 ± 12	55 ± 14
	3rd phase	50 ± 10	50 ± 7	54 ± 11	54 ± 12
	4th phase	44 ± 11	49 ± 8	46 ± 9	47 ± 11

*No significant difference was found between pre- and post-test both for training and control groups*.

Neither stretch of the tendon (Pre: 33 ± 13 mm vs. Post: 26 ± 6 mm) in the braking phase (1st and 2nd phases) nor shortening of the tendon (Pre: −28 ± 11 mm vs. Post: −38 ± 13 mm) in the propulsive phase (3rd and 4th phases) differed between the Pre-test and Post-test sessions of the training group. In contrast, shortening velocity of the tendon during the 3rd phase was significantly faster in the Post-test than in the Pre-test (Figure 4B). The control group displayed no differences between the Pre-test and Post-test sessions relating to stretch (Pre: 21 ± 6 mm vs. Post: 23 ± 6 mm), shortening (Pre: −28 ± 11 mm vs. Post: −27 ± 7 mm), or velocity of stretch and shortening (1st phase: 206 ± 53 mm/s [Pre], 182 ± 61 N mm/s [Post], 2nd phase: 64 ± 45 mm/s [Pre], 85 ± 42 mm/s [Post], 3rd phase: −76 ± 20 mm/s [Pre], −85 ± 60 mm/s [Post], and 4th phase: −177 ± 81 mm/s [Pre], −164 ± 40 mm/s [Post]) of the tendon.

### EMG activities during DJ

**Figure 5:**
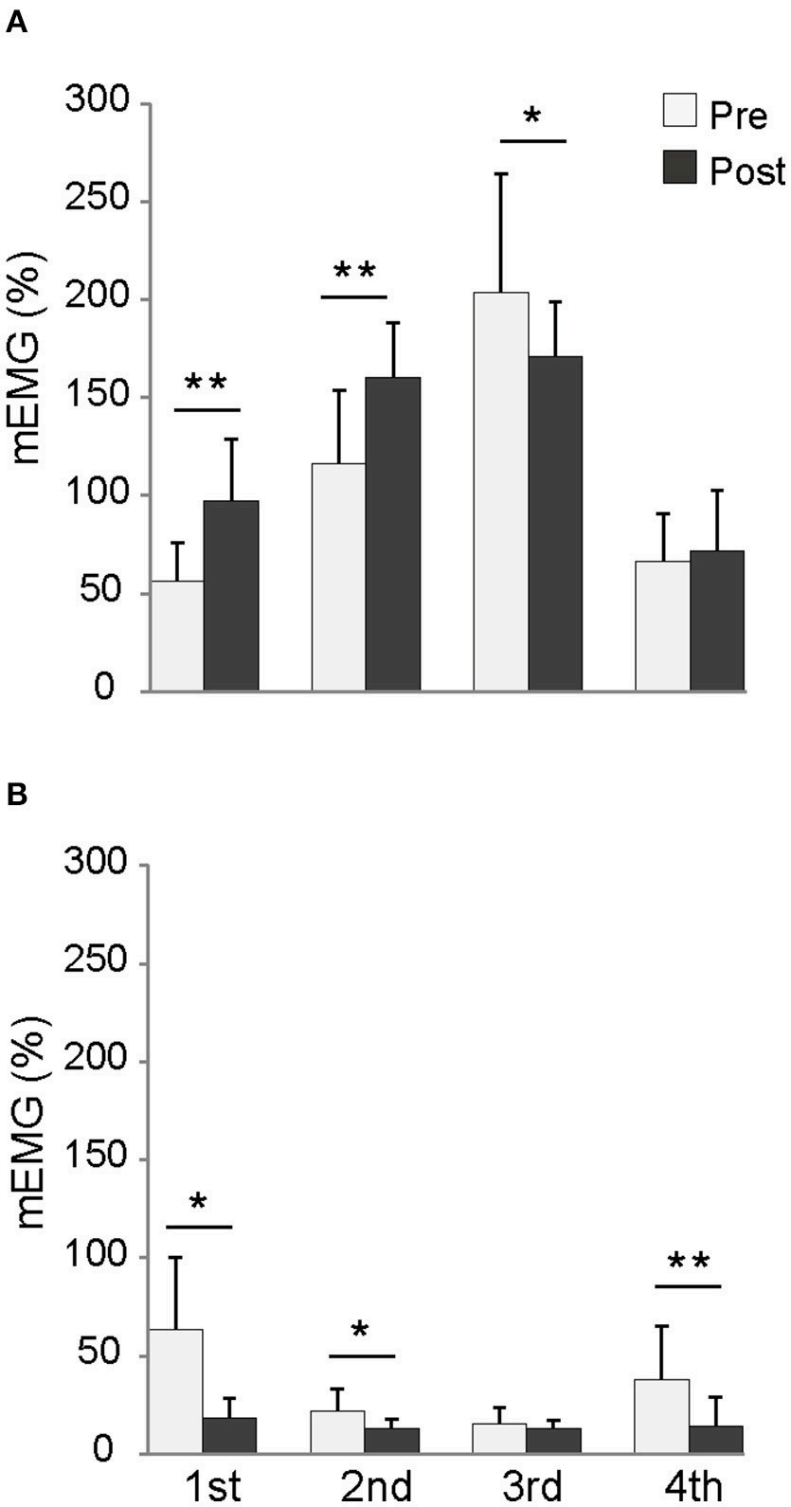
**mEMG of the triceps surae (A)** and tibialis anterior **(B)** during each phase of depth jump for the training group. mEMG: EMG value normalized with that during maximal isometric plantar flexion or dorsiflexion. ^*^p < 0.05, ^**^p < 0.01.

Figure 5 shows mEMGs of the triceps surae and tibialis anterior during DJ for the training group. The mEMGs of the triceps surae during the 1st and 2nd phases were significantly higher in the Post-test than in the Pre-test. In contrast, mEMG of the triceps surae during the 3rd phase was significantly lower in the Post-test than in the Pre-test. The mEMGs of the tibialis anterior during the 1st, 2nd, and 4th phases were significantly lower in the Post-test than in the Pre-test. For the control group, no differences between the Pre- and Post-test sessions were observed for mEMG of the triceps surae (1st phase: 49 ± 16% [Pre], 53 ± 23% [Post], 2nd phase: 88 ± 34% [Pre], 96 ± 36% [Post], 175 ± 45% [Pre], 158 ± 25% [Post], 75 ± 16 [Pre], 56 ± 27 [Post]) and tibialis anterior (1st phase: 53 ± 23% [Pre], 43 ± 22% [Post], 2nd phase: 29 ± 24% [Pre], 20 ± 14% [Post], 3rd phase: 15 ± 8% [Pre], 13 ± 5% [Post], 4th phase: 24 ± 21 [Pre], 23 ± 17 [Post]).

## Discussion

Impulse significantly increased in the training group, indicating that the plyometric training adopted in the present study improved the SSC exercise performance. Contact time with the force plate shortened and the averaged reaction force during the 2nd phase (latter half of braking) and 3rd phase (initial half of propulsion) increased. Therefore, the increase in impulse was not attributable to prolonged force production but was more probably due to the enhancement of plantar flexion torque around the transition from ankle dorsiflexion to plantar flexion. Maximal static plantar flexion torque did not increase significantly after the plyometric training. Therefore, the increase in reaction force during the 2nd and 3rd phases of DJ could not be explained by any increase in maximal strength of the agonists. It is more likely that the increase in reaction force is due to favorable changes in muscle–tendon behavior of the agonist during DJ. After the training sessions, fascicles were contracted isometrically over the 2nd and 3rd phases. Under this condition, the muscle could generate higher force, and stretch–shortening of the tendon became more influential. These changes were likely to be achieved through changes in tendon stiffness and neuromuscular activity of the agonists. These data support our hypothesis. Furthermore, reduced neuromuscular activity of the antagonist during the 2nd phase of DJ appears to play an important role in performance improvement.

### Possible mechanisms that increase reaction force during the 2nd and 3rd phases of DJ

In the 2nd phase, changes in force generated by both antagonists and agonists could have had an influence on increased average reaction force. Reduced activation level of the tibialis anterior could have increased net ankle plantar flexion torque, thereby enhancing the reaction force at the foot. With no change observed in averaged length or shortening velocity of fascicles, the triceps surae exhibited greater mEMG. These findings suggest that the triceps surae exerted greater force than that before the intervention. The reason that no change was observed in fascicle length or contraction velocity despite the increase in muscle activation is probably related to an increase in Achilles tendon stiffness. Regarding the force–length relationship of a tendon, a stiffer tendon resulted in the less fascicle shortening when the muscle was generating greater force. This has an advantageous effect in the force–velocity relationship, and the overall force increased in the output.

In the 3rd phase, the increase in averaged reaction force appears to be attributable to changes in muscle–tendon behavior of the agonists. The mEMG of the tibialis anterior remained unchanged in the 3rd phase. In contrast, fascicle shortening velocity of the medial gastrocnemius decreased to the point where contraction changed from concentric to isometric contraction. With fascicle length unchanged and mEMG activity lowered, the increase in the averaged reaction force was probably due to the decrease in fascicle contraction velocity. Simultaneously, shortening velocity of the tendon increased. Even though tendon elongation in the braking phase did not change as a result of the training, tendon stiffness and peak reaction force increased. This implies that the amount of elastic (potential) energy stored in the tendon prior to the propulsion phase (the beginning of the 3rd phase) was increased after the training. Transformation of this extra elastic (potential) energy into kinetic energy has been responsible for faster tendon shortening during the 3rd phase. Elastic recoil of the tendon counters muscle shortening since it was positioned as a series elastic component within the muscle. Thus, even though force produced by the muscle was increased, the fact that the fascicle did not shorten might be attributable to the stronger elastic recoil of the tendon in series. Given that mEMG of the triceps surae of the training group decreased significantly in the 3rd phase, it is possible that the subjects in the training group have managed to increase force production of the agonists by decreasing neuromuscular activation. This also would achieve a contraction mode better geared to generating force.

In summary, the kinematic changes observed in the medial gastrocnemius indicate that muscle–tendon interaction during SSC works more functionally to achieve better exercise performance. Generation of a high muscle force before the propulsive phase resulted in stretching of the stiffer tendon. Consequently, once the propulsive phase began, neuromuscular activation could be reined in to prevent sudden concentric muscular contraction (“concerted contraction”: Hof et al., 1983) while the quick elastic recoil of the tendon would become more influential.

### Reasons that the reaction force during the 1st and 4th phases of DJ did not increase

In the 1st phase, no change was observed in the reaction force at the foot. Reduced neuromuscular activation of the tibialis anterior and an increase in mEMG of the triceps surae could have contributed to an increase in the net plantar flexion torque and to the reaction force at the foot. However, eccentric contraction of triceps surae fascicle observed before training changed to concentric contraction after training, diminishing the degree of force that the muscle group could generate. We should note that muscle–tendon behavior during the 1st phase should not be associated with tendon stiffness, because the tendon stiffness was determined under a relatively high-intensity condition (>50% maximal voluntary contraction). Consequently, net plantar flexion torque and reaction force at the foot may have remained unchanged after the training period.

In the 4th phase, no change was observed in averaged reaction force. Neuromuscular activation of the antagonistic tibialis anterior decreased in the Post-test. Because the activity of that muscle in the Pre-test was observed just prior to leaving the force plate, it is most likely to have resulted in dorsiflex of the foot away from the plate (see Figure 2). Therefore, the observed change would have had little effect on increasing reaction force.

### Single-session practice vs. long-term training

Hirayama et al. (2012) reported that a single practice session changed muscle–tendon behavior during a counter-movement jump, leading to enhanced performance. Single-session practice (Hirayama et al., 2012) and long-term training (the present study) are similar in that there was no increase in neuromuscular drive of agonists observed during the propulsive phase that could have contributed directly to enhancing generation of the agonists force. In contrast, such a change in neuromuscular drive was observed during the braking phase in both studies. Based on these facts, we speculate that the sufficient activation of the agonists during the braking phase determines how effectively the muscle–tendon interaction could function. The major difference between the two studies is the increase in tendon stiffness found in the present study but not in that by Hirayama et al. (2012). In the present study, elastic recoil of a stiffer tendon would inhibit shortening of the fascicles, keeping the latter in the more powerful region of the force–velocity relation of muscle, thus leading to an increased shortening velocity of the tendon. Consequently, these facts suggest that the adaptation mechanism as a result of long-term training partly differs from that of a single-session practice.

### Influence of tendon stiffness on SSC exercise performance

Previous studies have failed to reach consensus regarding the contribution of an increase in tendon stiffness to SSC exercise performance (Kubo et al., 2007b; Wu et al., 2010; Houghton et al., 2013). The present findings suggest that an increase in tendon stiffness as well as alteration in neural activity after the longitudinal plyometric training play an important role in making muscle–tendon interaction more functional, resulting in successful SSC exercise performance. Although the negative effect of higher tendon stiffness on SSC exercise performance has been suggested in cross-sectional studies (Kubo et al., 1999, 2007a), the present study strongly suggests that the increase in tendon stiffness commensurate with the increase in the muscle force exerted during SSC exercise, leading to the SSC performance improvement.

### Concepts of performance enhancement in SSC exercise

An MTU exhibits greater power or work in the SSC than in a concentric contraction without prior stretching (denoted as “pure concentric”). The mechanism for this has been explained in various ways; stretch reflex (Komi, 2000), potentiation of the contractile component (also known as residual force enhancement) (Ettema et al., 1992), time available for active state development (also known as pre-activation) (Bobbert and Casius, 2005), utilization of elastic energy (Sugisaki et al., 2005), and muscle–tendon interaction (Kawakami and Fukunaga, 2006). The stretch reflex and potentiation of the contractile component can be ruled out since the fascicle was not lengthened in the Post-test of our training group. The mechanism of how performance is enhanced via the SSC appears to vary even within a given individual.

### “Joint stiffness”: an index of braking kinetics

Joint stiffness (determined from the angle-torque relation during braking movement) has been considered to be related to SSC exercise performance, but the underpinning mechanisms are poorly understood. Previous studies have shown that joint stiffness increases after plyometric training (i.e., the joint becomes “stiffer”), leading to improved performance of SSC exercise (Toumi et al., 2004; Kubo et al., 2007b). When used to calculate index of joint stiffness from the angle-reaction force relation during braking phase, data from our study revealed an increase in joint stiffness index in the training group (Pre: 2392 ± 327 N/rad, Post: 3724 ± 936 N/rad). The mechanism behind the increase in the joint stiffness index appears to be due to greater force of the agonists and less force in the antagonists during the braking phase as well as an increase in tendon stiffness. All these factors were regarded as being involved in the mechanism to improve SSC exercise performance. This increase in joint stiffness could thus serve as one index to reflect a change in the mechanism of improved performance of the SSC exercise.

### Limitations

The MTU observed in the present study had a relatively long series elastic component. Arakawa et al. (2010) have shown that the mechanism for enhancing performance by SSC may differ between an MTU with a long series elastic component and one with a short series elastic component. Hence, we speculate that the present findings may not apply to the gluteus maximus, which has little tendon, and may only partially explain the mechanisms of plyometric training-induced performance enhancement of muscle with a short series elastic component, such as the vastus lateralis. Ishikawa et al. (2006) reported that the fascicles and tendons of the vastus lateralis behaved in a stretch–shortening manner during multi-joint depth jumping. They observed that, when subjects intended to jump higher, the EMG amplitude of the vastus lateralis during the late braking phase and the shortening velocity of the tendon tissue increased. These findings are similar to those of the present study, and could support our speculation.

In addition, an assumption regarding the fascicle behavior should be noted. In both our study and the one by Hirayama et al. (2012), behavior of a fascicle from the medial gastrocnemius was assumed to represent that of the entire triceps surae. The medial and lateral gastrocnemii and the soleus each have their own physiological and morphological characteristics. In contrast, the medial gastrocnemius and the soleus have been shown to behave in a synchronized manner during concentric and eccentric movements (Chino et al., 2008) and SSC actions (Sakuma et al., 2012). Based on these facts, the behavior of the medial gastrocnemius represents fascicles of the soleus during a movement with only an ankle joint would not be vastly different from the actual behavior. The triceps surae includes the lateral gastrocnemius. To the best of our knowledge, there have been no reported studies of the behavior of the fascicles of the lateral gastrocnemius during SSC action; however, this muscle represents only about 12% of the physiological cross-sectional area of the triceps surae (Albracht et al., 2008). Thus, the mechanisms that we report in the present study would explain the major adaptation scenario of the triceps surae. The plantar flexors include other muscles, and future investigation of how each of these muscles behave might further our detailed understanding of the mechanism of SSC performance.

This study was conducted with a relatively small sample size. In addition, we were unable to obtain the ankle joint angle during DJ from three subjects in the training group and one subject in the control group. This probably reduced our statistical power for detecting marginal changes in DJ kinematics in the training group (such as the fascicle velocity during the second phase). Future studies using a larger sample size are needed to confirm the findings.

In this study, the training program did not progress in volume or intensity through the training period. This lack of progression may have resulted in the increase in muscle strength being non-significant. Even if the static muscle strength had increased after a progressive training program, the changes in the fascicle behavior observed during the braking phase (i.e., eccentric contraction becoming concentric contraction) would still have been observed. In the propulsion phase, subjects in the training group exhibited reduced EMG activity of the triceps surae. Thus, the changed behavior of the fascicle would have been observed in this phase as well.

## Conclusions

A 12-week regimen of plyometric training has been shown to improve SSC exercise performance by the optimization of muscle–tendon behavior of the agonists, associated with an alteration in the neuromuscular activity during SSC exercise and an increase in tendon stiffness. Furthermore, a decrease in the neuromuscular activity of the antagonist during the braking phase appears to play an important role in this improvement.

## Author contributions

Conceived and designed the experiments: KH and YK. Performed experiment: KH, SI, NI, and AY. Analyzed data: KH and SI. Interpreted results of research: KH, SI, NI, AY, RE, and YK. Drafting manuscript and prepared tables and figures: KH. All authors edited, critically revised paper and approved final version of manuscript, and have agreed to be accountable for all aspects of the work related to its accuracy and integrity.

### Conflict of interest statement

The authors declare that the research was conducted in the absence of any commercial or financial relationships that could be construed as a potential conflict of interest.
